# Personal Matters

**Published:** 1855-12

**Authors:** 


*Personal Matters.*—We are not of that class who condemn all controversies, either in medicine or literature. On the contrary, when conducted in a liberal spirit and in accordance with appropriate rules, they sharpen the intellects of the writers, enliven the minds of the readers, and often elicit new thoughts which result in the extension of the boundaries of knowledge. To accomplish these ends, however, the controversialist must be actuated by a liberal spirit, and a strict sense of justice. The first will always cause him to treat his opponent kindly, and the second to represent all his sentiments truly. Those who take the trouble to read the editorial articles of this Journal, are aware that a controversy has been maintained during the last few months, with one of the editors of the *Peninsular Journal*, concerning certain matters wherein the Medical Department of the University of Michigan claimed to be in advance of other schools.

Throughout this controversy we have wholly refused to reply to mere personal charges or allusions; we have strictly adhered to the legitimate topics involved in the controversy, and that we might represent the positions and sentiments of our opponent fairly and truly, we have been careful to quote, in his own language, every position and sentiment which had been made the subject of comment and criticism. When we alluded to the requirements of the Michigan school, we took the pains to quote them verbatim, directly from their last Annual Announcement. When a single quoted paragraph was charged with having been separated from its proper connections and its meaning thereby perverted, we promptly requoted it together with all that preceded and followed, to which it could claim any relationship; thereby enabling our readers to judge for themselves. Nor was this all. For when the only *harsh* word we had used, was complained of and misrepresented, we cheerfully explained the true sense in which we had used it, thereby placing it in a light which should have satisfied the most fastidious We pursued this course, not because our opponent was an old personal friend for whom we entertained special sentiments either of fear or favor, but simply because it was in accordance with our sense of propriety and our long established rules of action.

We regret to say that the editor of the *Peninsulur Journal*, Dr. A. B. Palmer, has seen fit to pursue a widely different course. Throughout the controversy, when alluding to the requirements of a rival school, instead of qnoting fairly and honorably from its published rules, he has conveyed false impressions by partial statements; arrogantly assumed the position of censor and charged the Faculty with delinquency in the performance of their public duties; and has well nigh exhausted the vocabulary of opprobrious terms, not to heap upon us, but to charge us with having used towards him. We had hoped that a persistent refusal, on our part, to reply to these personalities would eventually constrain our opponent to abandon so illiberal a course of conduct. In the December number of his Journal, however, while pretending to close the controversy, he has substantially repeated the most offensive of his personal charges, and with more arrogance than good taste, parades our courteous explanation of the sense in which we had used the word "falsehoods," as evidence of our shame and humiliation. It is an old maxim, that there is a point beyond which forbearance cases to be a virtue. We have now come precisely to that place. In the course of this controversy, Dr. Palmer has distinctly informed his readers, that we had used "*such* an array of *unmanly* evasions, *untruthful* statements, *false* accusations, and *bitter* bursts of feeling," as to utterly astonish him.

He has told them that we used "*false* and *abusive* language;" that we were "his *unjust* accuser—nay *his bitter traducer.*"

He charged us with having "*ignored*" the subject of preliminary education. And in his last article he again gives his readers to understand that we had previously passed "to the *extreme of rude courtesy* and *reckless misstatements*;" that we had accused him of "*falsehoods*, and *such other grossly unbecoming charges*," &c., &c. These are all strictly personal accusations, directly calculated to influence individual character; and we now call upon the editor of the *Peninsular Journal* either to retract every one of themor place the *proof* of their correctness before his readers. We do not ask for renewed assertions on his part; but if we have used "*unmanly* evasions, *untruthful* statements, and "*false* accusations," our language containing them is on record.

Let him place *that language* before his readers. If we have "unjustly accused" and "bitterly *tradnced*," him, the proof is on record. Let him place it before his readers. If we have "ignored" preliminary education—if we have exhibited the "extreme of *rude discourtesy* and reckless misstatements"—if we have accused him of "falsehoods, and such other *grossly* unbecoming charges," let our language, containing these things be placed before *his* readers, verbatim et literatim, and we will abide their judgement. He has made the charges against us to his own readers, and we have a right to demand that he now place before them the *proof* or an explicit retraction. When he honorably complies with this demand, we will reciprocate the compliment he has paid us in the following sentence, from his last article.

He says: "Our excitable neighbor has learned wisdom from experience, and appears to have come to the conclusion that a degree of discretion is the better part of valor." We are certainly glad that our friend has discovered evidence of this in us. To learn *wisdom* by experience, that me might thereby exhibit an increase of discretion or good judgement, has been, with us, one of the leading objects of life. We only regret that we have not discovered greater progress in the same direction, on the part of our Michigan neighbor.

